# Protocol for the establishment and morphological characterization of long-term cultivated murine cerebral organoids

**DOI:** 10.1016/j.xpro.2025.104324

**Published:** 2026-01-09

**Authors:** Issam El-Debs, Michael R. Knittler, Thomas C. Mettenleiter, John O. Mason, Julia Sehl-Ewert

**Affiliations:** 1 Department of Experimental Animal Facilities and Biorisk Management, Friedrich-Loeffler-Institut, Greifswald-Insel Riems, Germany; 2 Institute of Immunology, Friedrich-Loeffler-Institut, Greifswald-Insel Riems, Germany; 3 Friedrich-Loeffler-Institut, Greifswald-Insel Riems, Germany; 4 Institute for Neuroscience and Cardiovascular Research, University of Edinburgh, Edinburgh, Scotland

**Keywords:** Microscopy, Neuroscience, Organoids

## Abstract

Murine cerebral organoids provide a rapid and reproducible *in vitro* system that recapitulates key aspects of neurogenesis. While human cerebral organoid protocols are well established, methods for non-human models remain limited. Here, we present a protocol for generating long-term cultured murine cerebral organoids from E14.5 embryonic stem cells (ESCs). We describe steps for generating mature organoids, followed by histological processing including paraffin embedding and microtome sectioning. We then detail procedures for characterizing murine cerebral organoids through H&E staining and immunofluorescence techniques.

## Highlights

• Optimized B27-guided protocol for long-term murine cerebral organoids
• Stepwise maturation from neuroepithelial rosettes to cortical-like layers
• Serial sectioning with histology and IF enables structural characterization
• Murine cerebral organoids as a model to complement *in vivo* studies

## Abstract (editor highlights)

Publisher’s note: Undertaking any experimental protocol requires adherence to local institutional guidelines for laboratory safety and ethics.

## Graphical abstract

**Figure:**
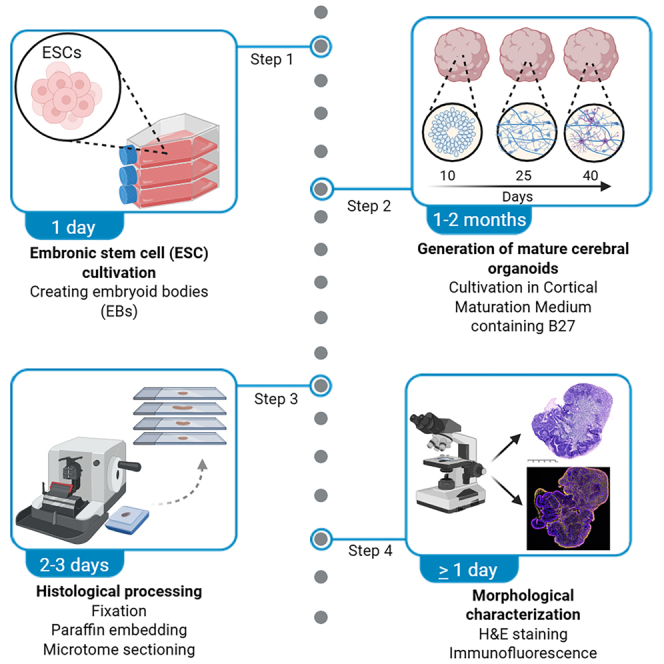

## Before you begin

All procedures for cultivating embryonic stem cells (ESCs) and generating murine cerebral organoids (COs) must be performed under sterile conditions in a Class II biological safety cabinet. Organoid culture requires an orbital shaker placed in a humidified incubator at 37°C with 5% CO_2_. This protocol employs the E14tg2a mouse ESC line (derived from 129/Ola mouse blastocysts).⁠^1^ These cells are pluripotent and efficiently differentiate toward neuroectodermal lineages under defined, serum-free culture conditions.

Before starting, prepare all media and reagents as listed in “materials and equipment”. Some reagents can be prepared in advance and stored as indicated; others must be freshly prepared immediately before use. Complete information, including catalog numbers and storage conditions, is provided in the key resources table.

Ensure that all culture plasticware (e.g., low-attachment 96-well plates), media supplements (e.g., B27), and handling tools are available and equilibrated to room temperature or 37°C as appropriate. Accurate timing, consistent cell handling, and gentle pipetting during embryoid body (EB) formation are critical for reproducibility and organoid viability.

### Innovation

Existing protocols for murine cerebral organoids are often constrained by short culture durations, which limit structural maturation and reduce their utility for downstream analyses. A central innovation of the present protocol is the ability to maintain murine cerebral organoids over substantially extended time periods, thereby enabling the development of more mature cytoarchitectural features. The optimized culture workflow reliably supports long-term viability and sustained tissue organization without requiring specialized equipment or genetic modifications.

A further key advancement is the comprehensive tissue processing strategy introduced here. Entire organoids are embedded, sectioned across multiple planes, and examined histologically at different tissue levels. This approach provides a more complete representation of organoid cytoarchitecture than surface-focused or single-plane analyses, allowing detailed assessment of global morphology, cortical layer–like features, and overall structural integrity.

Finally, this protocol establishes a species-specific murine organoid platform that could complement existing mouse in vivo models. Depending on the research question, this system can reduce or refine animal use by providing an experimentally accessible, physiologically relevant in vitro counterpart. Together, these methodological innovations expand the applicability of murine cerebral organoids for mechanistic and comparative studies.

## Key resources table

**Table:** 

REAGENT or RESOURCE	SOURCE	IDENTIFIER
**Antibodies**		

BCL11b/CTIP2 (1:1000)	Invitrogen/rabbit	Cat# PA5-117429
Cleaved caspase-3 (1:800)	Cell Signaling Technology/rabbit	Cat# 9661
GFAP (1:400)	Thermo Fisher Scientific/chicken	Cat# PA-110004
Nestin (1:1000)	Invitrogen/chicken	Cat# PA5-143578
NeuN (1:200)	SYSY/guinea pig	Cat# 266004
Ki-67 (1:100)	Invitrogen/rabbit	Cat# MA5-14520
Olig2 (1:50)	Abcam/rabbit	Cat# 109186
PAX6 (1:100)	Invitrogen/rabbit	Cat# 42-6600
Reelin (1:100)	Invitrogen/rabbit	Cat# PA5-78413
SATB2 (1:1000)	Invitrogen/rabbit	Cat# ZE4319632A
SOX1 (1:1000)	Abcam/rabbit	Cat# 87775
SOX2 (1:100)	Invitrogen/rat	Cat# 14-9811-82
TBR1 (1:500)	Invitrogen/rabbit	Cat# YF3956298A
TBR2/EOMES (1:500)	Invitrogen/rabbit	Cat# 720200
TUJ1 (1:200)	Invitrogen/mouse	Cat# MA1-118
Anti-mouse Alexa Fluor 488 (1:200)	Invitrogen/Goat	Cat# A11001
Anti-rabbit Alexa Fluor 488 (1:200)	Abcam/Goat	Cat# AB150077
Anti-chicken Alexa Fluor 568 (1:200)	Thermo Fisher Scientific/Goat	Cat# 2079349
Anti-guinea pig Alexa Fluor 647 (1:200)	Thermo Fisher Scientific/Goat	Cat# A21450
Anti-rat Alexa Fluor 633 (1:200)	Invitrogen/Donkey	Cat# A21094

**Biological materials**		

Mouse embryonic stem cells (E14.5)	Hooper et. al.^⁠^^1^	Provided by the laboratory of John O. Mason from the University of Edinburgh, UK.

**Chemicals**		

β-Mercaptoethanol	Fisher Scientific	Cat# 21985023
Ammonium chloride (NH_4_Cl)	Roth	Cat# P726.1
Bovine serum albumin (BSA)	MP Biomedicals	Cat# 08810061
Chloral hydrate	Roth	Cat# K318.1
Citric acid monohydrate	Roth	Cat# 3958.1
Copper(II)-sulfate (CuSO_4_)	Roth	Cat# CP86.1
Embryonic stem cell FBS	Fisher Scientific	Cat# 16141079
Eosine Y	Sigma	Cat# E−4382
Ethanoic acid	Roth	Cat# 3738.2
Hematoxylin	Roth	Cat# 3816.1
Corning Matrigel Growth Factor Reduced (GFR) Basement Membrane Matrix	Sigma-Aldrich	Cat# CLS354230-1EA
DMEM/F-12, GlutaMAX Supplement	Fisher Scientific	Cat# 31331093
Dimethyl sulfoxide (DMSO)	PAN Biotech	Cat# P60-36720100
Dipotassium phosphate dihydrate	Roth	Cat# 10028-24-7
ESGRO Recombinant Mouse LIF Protein (10^6^ units)	Sigma-Aldrich	Cat# ESG1106
Embryonic stem-cell FBS	Fisher Scientific	Cat# 16141079
Entellan	Merck	Cat# 1.07961.0100
Gelatin	Roth	Cat# 273327122
GlutaMAX Supplement	Fisher Scientific	Cat# 35050061
HOECHST 34580	Thermo Fisher Scientific	Cat# 2158924
Inhibitor of WNT Production-2 (IWP2)	Merck	Cat# I0536-5MG
Isopropanol	Roth	Cat# 6752.5
KnockOut serum replacement	Gibco	Cat# 10828028
MEM solution from non-essential amino acids (100X)	FisherScientific	Cat# 11140050
Mikrozid	Schülke	Cat# 1604009
N-2 Supplement (100x)	Invitrogen	Cat# 17502001
MEM NEAA (100X)	Gibco	Cat# 11140-050
NeuroCult SM1 Neuronal Supplement (50X)	STEMCELL Technologies	Cat# 05711
Paraffine	Merck	Cat# 1.07164.2504
Paraformaldehyde	Roth	Cat# 30525-89-4
Phloxine B	Sigma	Cat# P-2759
Potassium alum (KAl(SO_4_)_2_·12H_2_O)	Roth	Cat# P724.1
Potassium chloride	Roth	Cat# 7647-14-5
Sodium iodate	Merck	Cat# 1.06525.0100
Sodium Pyruvate (100 mM)	Fisher Scientific	Cat# 11360070
Triton X-100	Sigma-Aldrich	Cat# 102348717
TrypLE Express Enzyme (1x), no phenol red	Fisher Scientific	Cat# 12604021
Xylene	Roth	Cat# 1330-20-7

**Equipment**		

12 Well Cell Culture Plate, sterile	Corning Incorporated costar	Cat# 3513
Brightfield microscope (inverted)	Carl Zeiss Microimaging	Cat# 451485
Biopsy cassette	Roth	Cat# 3941263169
Cell chamber Neubauer improved	Carl Roth	Cat# T729.1
Cell culture flask (T25/T75) - vented cap, sterile	NP Green tip	Cat# 06-695-5300
Class II biological safety cabinet, sterile	Labogene, SCANLAF	Cat# 05 180 456
CO_2_ incubator, sterile	Sanyo Electric Co.	Cat# MCO-19AIC
Corning CoolCell (“Mr. Frosty”)	Corning	Cat# 432000
Cooling plate for microtome sectioning	Medite	Cat# 901 679 0911
Falcon Tubes (15 and 50 mL), sterile	Sarstedt	Cat# 62.554.502
Filter (0,22μm), sterile	Millex GP	Cat# SLGP033RS
Filter paper	Roth	Cat# 660P
Fisherbrand Transfer pipettes, sterile	Fisher Scientific	Cat# 13469108
Grease pencil	Vector Laboratories	Cat# H-4000
Heating unit	Medite	Cat# 2411414
IKA shaker	VWR	Cat# 444-0665
Incubator (37°C, 5% CO_2_)	Sanyo	Cat# 11040395
Laboratory freezer (−80°C)	Eppendorf	Cat# 1005-8397-0312
Laboratory refrigerator (4°C/–20°C)	Liebherr	Cat# 32.635.442.9
Laboratory scale	Sartorius AG Germany	Cat# 26609647
Lab-pH-Meter 766, Calimatic	Knick Elektronische Meßgeräte GmbH & Co	Cat# 1276250
Razor blades	Feather	Cat# 230704308
Vacuum tissue infiltration machine ASP 300S	Leica	Cat# HIS 00029
Magnetic stirrer	Heidolph Instruments GmbH & Co.KG	Cat# 200 652765
Orbital shaker	Heathrow Scientific	Cat# HSB04971
Paraffin pouring system (dispensing unit)	Medite	Cat# 902 002 0911
Paraffin stretching bath	Medite	Cat# 904 844 0811
Petri dish (100mm x 20mm)	CORNING	Cat# 353003
Pipette (1, 20, 200, and 1,000 μL)	Eppendorf ResearchPlus	Cat# 300436
Pipette (5 mL)	Costar	Cat# CLS7045-200EA
Pipetboy	Eppendorf Easypet	Cat# 2069422
PrimeSurface 3D culture: Ultra-low Attachment Plates: 96 well, U bottom	MoBiTec GmbH	Cat# MS-9096UZ
Rapid slide dryer	Medite	Cat# 901 581 0911
Rotation Mikrotom Hyrax M55	Zeiss	Cat# Hyrax M55 52292
Single-use syringe (25 mL)	BRAUN	Cat# 23D03C8
Slide stretching table	Medite	Cat# 905 296 0911
Shandon ImmuMount	Thermo Fisher Scientific	Cat# 10662815
SuperFrost-Plus Adhesion Microscope Slides	Epredia	Cat# J1800AMNZ
Turbo Cuisine - Steam cooker	Tefal	Cat# CY778830
Tissue Stainer	Medite	Cat# TST 44.000C
Water bath	Gesellschaft für Labortechnik mbH (GFL)	Cat# 1003

## Materials and equipment

### Preparation of solutions for the cultivation of murine ESCs

**Timing: 10 min**

Prepare the following sterile solutions (pre-warm to 37°C where indicated).

**Table:** Embryonic stem cell medium (ESC Medium)

Reagent	Final concentration	Amount [ml]
DMEM/F12/Glutamax (1X)	N/A	43
Sodium pyruvate	1mM	0,5
MEM NEAA (100X)	1mM	0,55
GlutaMax (100X)	1mM	0,5
β-Mercaptoethanol	0,1mM	0,1
Fetal bovine serum (FBS)	10%	5
Leukemia inhibitory factor (LIF)	1x10^6^ Units/ml	0,055
Total		50

Mix all components under sterile conditions. Store FBS at −20°C, all other reagents at 4°C. Store complete medium at 4°C up to 1 month.

***Note:*** Monitor medium color (Phenol Red). Warm pink-red = acceptable; orange/yellow = acidification/contamination → discard.

In case you plan to preserve cells, prepare the necessary cryopreservation solution for ESCs as follows.

**Table:** ESC freezing medium

Reagent	Final concentration	Amount [ml]
DMEM/F12/Glutamax (1X)	N/A	40
Fetal bovine serum (FBS)	10%	5
Dimethyl sulfoxide (DMSO)	10%	5
Total		50

Mix sterile. Store DMSO at RT, FBS at −20°C, DMEM at 4°C. Store complete medium at 4°C up to 1 month. For cryopreservation, aliquot 500 μL and freeze gradually at −80°C using a controlled-rate freezing container; store long-term at −80°C.

### Preparation of solutions for murine cerebral organoid generation

**Timing: 30 min**

**Table:** Knock-out serum replacement (KSR-) medium

Reagent	Final concentration	Amount [ml]
DMEM/F12/Glutamax (1X)	N/A	44
Sodium pyruvate	1mM	1,1
MEM NEAA (100X)	0,1mM	0,55
KnockOut™ serum replacement (KSR)	10%	5
β-Mercaptoethanol	0,1mM	0,114
Inhibitor of WNT Production-2 (IWP2)	2,5μM	5μl
Total		50

Prepare fresh under sterile conditions. Store KSR at −20°C, others at 4°C. Discard after use.

**Table:** Cortical Maturation Medium (CMM)

Reagent	Final concentration	Amount [ml]
DMEM/F12/Glutamax (1X)	N/A	44
GlutaMax (100X)	1mM	0,5
N-2 Supplement (100x)	1X	0,5
NeuroCult™ SM1 Neuronal Supplement (B27-equivalent, 50X)	1X	1
Total		50

Mix sterile. Store complete medium at 4°C for up to 1 month.

***Note:*** SM1 is functionally equivalent to B27.

### Preparation of buffers for hematoxylin and eosin staining

**Timing: 60 min**

Prepare Mayer’s hematoxylin and the eosin-phloxine solution as follows.

**Table:** Mayer’s hematoxylin

Reagent	Final concentration	Amount
Hematoxylin	0,1%	1g
Sodium iodate	0,02%	0,2g
Potassium alum	5%	50g
Chloral hydrate	5%	50g
Citric acid	0,1%	1g
Deionized water		1000ml

Dissolve sequentially with stirring; filter before use. The solution appears red-violet and can be reused. Store between 20°C–25°C.

**Table:** Eosin-Phloxine

Reagent	Final concentration	Amount
Eosin Y and Phloxine B are first mixed separately, then combined to prepare the eosin-phloxine solution.		
Eosin Y	1%	1g
Deionized water	N/A	100ml
Phloxine B	1%	1g
Deionized water	N/A	100ml
Esoin Y in deionized water	N/A	200ml
Phloxine B in deionized water	N/A	20ml
Ethanol	95%	1560ml
Glacial acetic acid	N/A	8ml
Total	N/A	1788ml

Mix until homogeneous; filter before use. Reusable. Store between 20°C–25°C.

### Preparation of solutions for immunofluorescence staining

**Timing: 1 h**

Prepare the following buffers and solutions required for immunofluorescence staining. Ensure all reagents are prepared fresh or stored as indicated.

**Table:** Permeabilization buffer

Reagent	Final concentration	Amount
Gelatin	N/A	0,5g
PBS 1X	N/A	125ml
Mix with stirring and heat at 55°C until dissolved. Then, add:		
Triton X-100	N/A	0,625ml
Total	N/A	250

Filter and adjust pH 7,4 with hydrochloric acid (HCl). Can be used immediately or stored at 4°C for 10 days.

***Note:*** Dilute 35% BSA with this buffer to 5% BSA (blocking buffer). For antibody dilutions, prepare 1% BSA in 1× PBS by diluting the 5% stock with PBS.

**Table:** Citrate buffer

Reagent	Final concentration	Amount
Citric acid monohydrate		2,1g
Deionized water		1000ml

Mix until homogeneous, adjust pH 6 with 2M sodium hydroxide (NaOH). Can be used immediately or stored at 4°C for long-term use.

**Table:** Washing buffer 2

Reagent	Final concentration	Amount
Copper(II)-sulfate (CuSO_4_)	10mM	0,4g
Ammonium chloride (NH_4_Cl)	50mM	0,65g
Deionized water		250ml

Mix until homogeneous. Ready to use. Store between 20°C–25°C for 1 month, protect from light.

**Table:** PBS 20X

Reagent	Final concentration	Amount
Disodium phosphate dihydrate	8mM	29,25g
Sodium chloride	137mM	160g
Potassium chloride	2,7mM	4g
Dipotassium phosphate dihydrate	2mM	4,9g
Deionized water		1000ml

Dissolve completely; adjust pH to 7.3 with NaOH/HCl. Dilute 1:20 to obtain 1× PBS. Store between 20°C–25°C.

## Step-by-step method details

### Preparation of murine ESCs

**Timing: 2 days**

This section describes ESC cultivation. Perform all steps under sterile conditions. Prior to seeding, coat flasks with 0.1% gelatin (the same gelatin used for the permeabilization buffer) to promote attachment.

1. Warm liquefied gelatin at 37°C for 10min.
2. Prepare 1% gelatin (1ml gelatin + 100ml of 1X PBS).
3. Sterile-filter through 0.22 μm.

***Note:*** Solution is viscous; load into a 25 ml syringe and filter slowly. Dilute to 0.1% (5 ml 1% gelatin + 45 ml PBS). Add 5 ml (T25) or 10 ml (T75) to coat the flask bottom.

***Note:*** Ensure full coverage to avoid uneven attachment.

4. Incubate the coated flasks 30min between 20°C–25°C.

***Optional:*** Shorter incubation may reduce attachment; more prolonged incubation has no adverse effect.

5. While flasks incubate, prepare the cells: If frozen at −80°C, thaw rapidly at 37°C with gentle swirling.
6. Transfer cells to a 15 ml tube with 5 mL pre-warmed ESC medium, centrifuge at 150x g, 5min (up to 250x g possible).
7. Discard supernatant carefully, resuspend the cell pellet in 10ml ESC medium.
8. After 30min of incubation, remove gelatin from the flask and seed the cell suspension.

**Figure 1:**
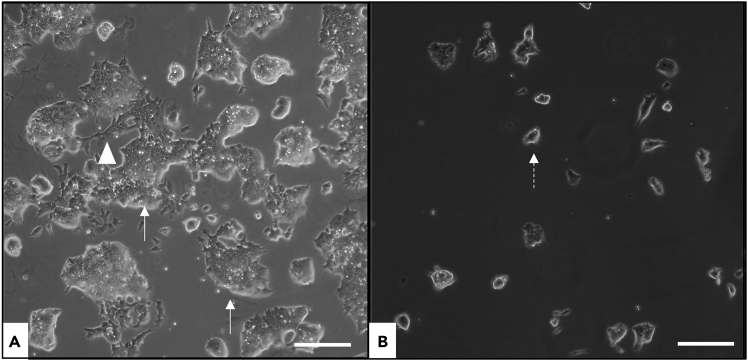
Representative images of embryonic stem cell (ESC) cultivation in medium supplemented with leukemia inhibitory factor (LIF) (A) Healthy ESCs form loosely organized adherent colonies (arrow) with cytoplasmic extensions (arrowhead). (B) Degenerating ESCs appear as small, round, non-adherent cells in suspension (dashed arrow). Scale bar: 200 μm.

***Note:*** Ensure confluency of at least 50% before freezing.

***Note:*** Assess viability and morphology using an inverted brightfield microscope. Non-viable cells: small, round, dark, non-adherent; viable ESC: phase-bright, adherent, amorphous colonies with cytoplasmic extensions (see Figure 1).

9. Incubate at 37°C, 5% CO_2_ for at least 12h.
10. Next day, assess cell health by evaluating overall morphology and adherence, replace with fresh ESC (medium with fresh ESC medium: 5ml T25/10ml T75).

**Figure 2:**
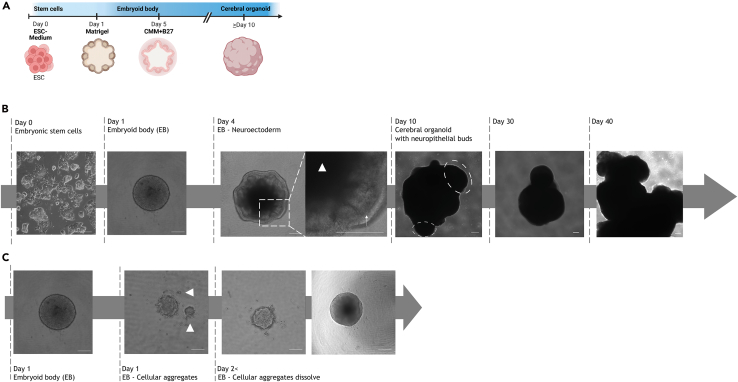
Brightfield images of embryoid body (EB) and cerebral organoid (CO) development (A) Timeline of CO generation: ESC seeding, EB formation within 24 h, Matrigel embedding, transfer to Petri dishes in cortical maturation medium (CMM) + B27 at day 5, and culture on an orbital shaker from day 10 onward. Created in BioRender. El-Debs, I. (2026) https://BioRender.com/t8h8ko1. (B) Representative images show ESC morphology, EB formation by day 1, emergence of neuroepithelial structures (arrow) and necrotic core (arrowhead) by day 4, and neuroepithelial buds (dashed circles) by day 10, indicative of cortical-like development. (C) Representative image of EB formation with occasional cellular aggregates (arrowheads) that may fuse or dissolve, forming a consistent EB after 2 days. Scale bars: 200 μm.

***Note:*** If cells are adherent with cytoplasmic extensions, EB formation typically begins within 24 h (Figure 2). Proceed directly to step 18 to initiate EB formation, or continue with cryopreservation (step 11).

11. Aspirate medium, wash 2x with 1x PBS.

**CRITICAL:** Remove FBS prior to detachment; serum inhibits TrypLE.

12. Add 1ml TrypLE, incubate 5min at RT (or until detached).

***Note:*** TrypLE stored at 4°C; commercial trypsin is acceptable (store −20°C; after thaw 4°C).

13. Confirm detachment microscopically.
14. Neutralize TrypLE with 9ml DMEM + FBS, then rinse the surface to dislodge cells.
15. Dilute cell suspension 1:10-1:20 with fresh ESC medium depending on confluency.

***Note:*** Passage cells at approximately 50%–70% confluence to maintain pluripotency. If cultures are <70% confluent, dilute at 1:5; avoid >90% confluence to prevent spontaneous differentiation.

16. Return to incubator (37°C, 5% CO_2_).
17. Next day (for freezing):
  a. Detach as in steps 11–14.
  b. Centrifuge 150x g, 5min, discard the supernatant, resuspend in 1ml pre-cooled freezing medium.
  c. Aliquot 500μl to cryovials, freeze gradually at −80°C.

***Note:*** Prior to cryopreservation, cells should be maintained at a confluency of approximately 50%–70%, as this range supports optimal viability after thawing. Performing an additional passage before freezing allows the cells to recover and adapt to the culture medium, thereby improving overall cell health and increasing the likelihood of successful post-thaw recovery.

### Development of EBs

**Timing: 20 min**

EBs are 3D ESC aggregates that later develop into COs once neuroepithelial buds emerge (approx. day 7–10). See Figure 2.⁠^2^

#### Day 0: Prepare a single-cell suspension

***Note:*** Prepare fresh KSR medium as described in the Materials and Equipment section.

18. At 70%–80% ESC confluency, aspirate medium and wash 2x with 1X PBS.
19. Discard PBS and add 1ml TrypLE.
20. Detach as in steps 11–14.
21. Add 5ml DMEM to neutralize TrypLE, transfer to a 15ml tube.
22. Centrifuge at 250x g, 5min.
23. Discard supernatant, resuspend in 5ml freshly prepared KSR medium.
24. Count viable cells:
  a. Mix 10μl Trypan blue + 10μl cells.
  b. Load 10μl on Neubauer counting chamber.
  c. Count viable (unstained) cells using a brightfield microscope or an automatic cell counter.
25. Dilute the cells in freshly prepared KSR medium to achieve a final seeding density of approximately 1000 cells per well (use 10 mL KSR medium per 96-well plate).

***Note:*** A seeding density of approximately 1000 cells/well ensures optimal organoid viability and survival. Deviations from this density, either lower or higher, may negatively affect organoid survival and growth.

26. Add IWP-2 (Inhibitor of WNT Production-2) to a final concentration of 2.5μM.
27. Seed around 1000 cells/well in 100μl of KSR into a 96-well U-bottomed plate.
28. Incubate at 37°C, 5% CO_2_ for 24h, then proceed with Matrigel embedding in step 29.

***Note:*** (Day 1), EBs typically appear as round, compact clusters with smooth translucent borders. Small clumps/debris may appear but usually dissolve over time and do not impair development (Figure 2).

***Note:*** (Day 1–3), EBs are undifferentiated aggregates (germ layer-like).⁠^3^ By day 4, early neuroepithelial structures emerge; The transition to CO-identity begins when neuroepithelial buds are visible (approx. day 7–10).⁠^4^

### Generation of mature murine cerebral organoids

**Timing: 30–60 days**

EBs are embedded in Matrigel; developing COs are cultured on an orbital shaker to enhance oxygen/nutrient diffusion. CMM using B27 supports robust growth, maturation, and neuroepithelial organization.

29. Day 1: Matrigel embedding of EBs.
  **CRITICAL:** Matrigel (−20°C) solidifies above 10°C, keep on ice at all times.
  a. Thaw Matrigel at 4°C for at least 10h on ice.
  b. Pre-cool 10μl pipette tips at −20°C for at least 15min.
  c. Add 200μg/ml of Matrigel (1μl per Well) directly to each EB well without removing the existing medium.
    **CRITICAL:** Bring the tip to the bottom into close proximity with the EB (slide along the wall until a slight bend) to ensure full EB coating and avoid hypoxic/uncoated zones (see Figures 3 and 4). If Matrigel begins to solidify prematurely, only partial coating will occur, or the material may solidify within the medium rather than around the EB, resulting in detached Matrigel fragments floating in the well. These fragments do not negatively impact organoid growth.
    **Figure 3:**
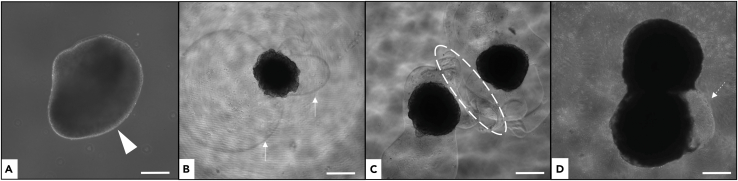
Impact of Matrigel polymerization on 10-day-old cerebral organoid (CO) differentiation Matrigel was added 24 h after EB formation and cultured for 4 days before transfer to Petri dishes. By day 10, COs developed neuroepithelial buds. (A) Proper Matrigel application with uniform surface coating and smooth morphology (arrowhead). (B) Premature polymerization causing incomplete coverage (arrows), disrupting neuroepithelial integrity. (C) Early solidification leading to entanglement between regions (circle), impairing growth. (D) Fusion of two COs (dashed arrow) due to premature solidification and insufficient separation. Scale bar: 200 μm.

**Figure 4:**
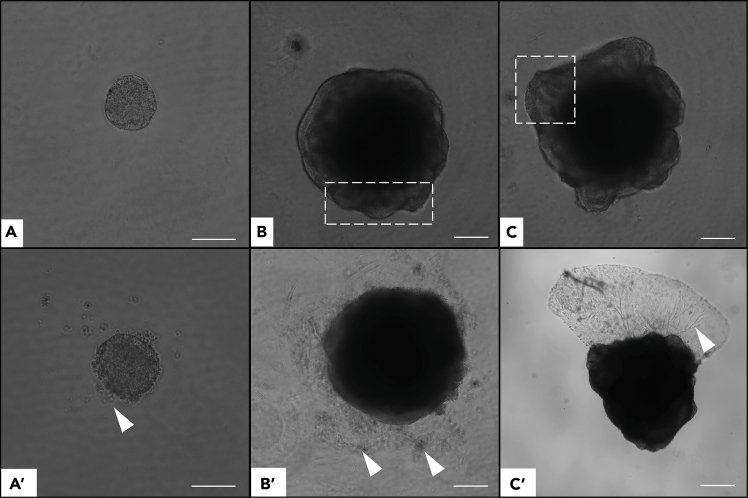
Brightfield comparison of well-formed versus defective cerebral organoids (COs) from embryoid bodies (EBs) (A) Day 2: EB forming a spherical neurosphere with uniform morphology. (A′) Incompletely formed EB with irregular aggregation (arrowhead). (B) Day 4: differentiating EB showing translucent neuroepithelial structures (box). (B′) EB lacking clear neuroepithelial features and surrounded by non-viable cells (arrowheads). (C) Day 5: expanding neuroepithelial regions (box). (C′) Day 10: CO with premature Matrigel solidification, disrupted coating, and disorganized migratory cells (arrowhead); neuroepithelial structures absent. Scale bars: 200 μm.
30. Incubate 4 days at 37°C, 5% CO_2_.

#### Day 2–4: Daily monitoring

***Note:*** Look for translucent neuroectodermal borders indicating neuroepithelial buds that later form neuroepithelial rosettes (Figure 2B). During this period, no medium change is required.

#### Day 5: Induction of cortical maturation

31. Place a sterilized orbital shaker in the incubator (37°C, 5% CO_2_).

***Note:*** Orbital motion promotes and improves nutrient/oxygen distribution.

32. Prepare fresh CMM as described in the materials and equipment section.
33. Supplement with 1X B27/SM1.
34. Add 10ml CMM to a 100mm Petri dish.

***Note:*** Organoids may fuse in the dishes (see Figure 3D). To prevent fusion, use 12-well plates and increase shaking to ≥50 rpm.

35. Transfer organoids (max. 12 per dish) with a sterile Pasteur pipette.

***Note:*** Alternatively, place one organoid per 12-/24-well to prevent fusion.

**Figure 5:**
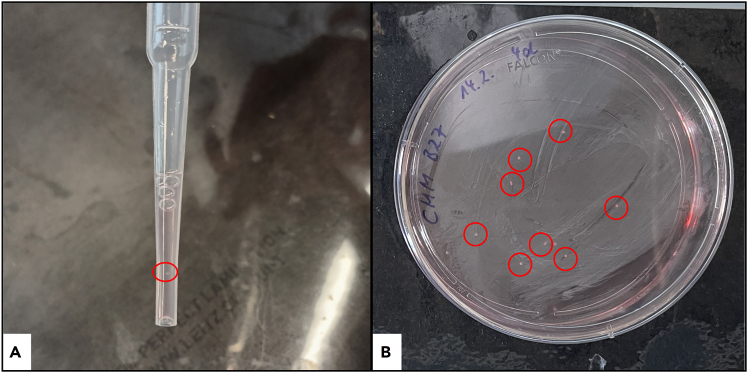
Representative images of cerebral organoid (CO) handling and culture A black sheet was placed beneath the dish to improve contrast. Circles mark individual organoids. (A) Day 5, COs (<500 μm) appear as small clusters and are collected using a Pasteur pipette. (B) Transferred COs in a 100-mm Petri dish with cortical maturation medium (CMM) + B27; approx. 8 organoids are evenly distributed across the dish.

**CRITICAL:** Early organoids (<20 days) are fragile and small (<400 μm). Use a black background under the dish to enhance visibility; confirm the organoid is inside the tip before release (Figure 5).

36. Incubate on shaker at 36–40 rpm, 37°C, 5% CO_2_.

#### Day 7+: Growth and maturation

37. Change medium every other day:
  a. Carefully aspirate 5ml from the edge.
  b. Replenish with 5ml fresh pre-warmed CMM.

***Note:*** Use a black sheet under the plate to avoid aspirating organoids.

38. Monitor development (Figure 2).
  a. Day 7–10: neuroepithelial buds visible.
  b. Size increases with maturation.

***Note:*** With B27/SM1, organoids typically reach approx. 1 mm by day 30 and >2 mm by day 40.

### Fixation and embedding of organoids

**Timing: 2 days**

This section describes the chemical fixation for downstream morphology, cell composition, and protein expression. Early organoids are small (<400 μm) and can be lost; older ones (>1 mm) are fragile. The size of early-stage organoids may complicate fixation and embedding. In contrast, more mature organoids (>1mm) are structurally fragile and prone to mechanical disruption, necessitating particularly careful handling.

39. Transfer up to 8 organoids into 1,5ml tubes containing 1ml of 4% paraformaldehyde (PFA) using a Pasteur pipette.

***Note:*** PFA is toxic; prepare/handle in a fume hood.

40. For better visibility, add 1-2 drops of Eosin-Phloxine; incubate 24h between 20°C–25°C.
41. Perform dehydration in the same tubes.
  **CRITICAL:** During exchanges, leave a small residual volume to avoid aspirating organoids. Dehydrate as follows:
  a. dH_2_O 1h.
  b. 50% isopropanol, 30min.
  c. 70% isopropanol, 30min.
  d. 80% isopropanol, 30min.
  e. 96% isopropanol, 30min.
  f. 99,8% isopropanol 2x30min.
  g. xylene 2x 30min.
  h. paraffine wax at 56°C for at least 12h.
    ***Optional:*** Early-stage organoids (up to day 20) are best processed manually. Larger organoids (> day 25; >1mm) may be processed on an automated tissue processor. Place organoids in biopsy cassettes lined with formaldehyde-moistened filter paper to minimize sample loss. Expect shrinkage during dehydration.
    Embedding (next day).
  i. Place the mold on a hotplate; add a small amount of molten paraffin.
  j. Center the organoid with forceps.
  k. Place the cassette lid and fill it to the brim with molten paraffin.
  l. Transfer to a cold plate and allow complete solidification.
42. Store FFPE blocks between 20°C–25°C until use.

### Microtome sectioning of FFPE organoids

**Timing: 30 min**

**Figure 6:**
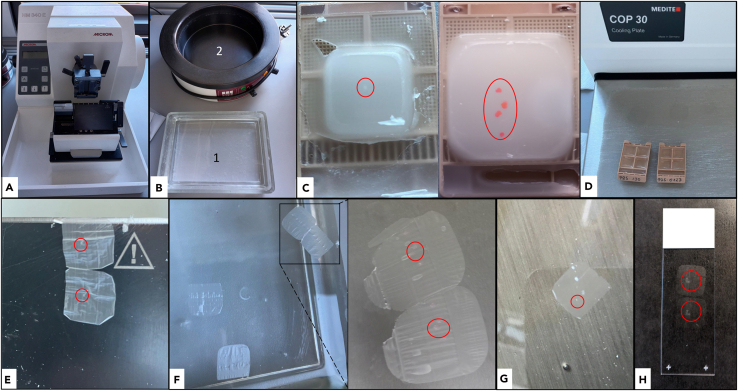
Workflow for microtome sectioning of FFPE organoids Red circles indicate the location of individual organoids. (A) Microtome apparatus used for sectioning. (B) Preparation of two water baths: one at room temperature (RT) and one at 40°C. (C) Visualization of organoids embedded in FFPE blocks. Organoids are nearly translucent without eosin pre-treatment (left), whereas eosin staining enhances visibility (right). (D) Cooling of the FFPE block on a cooling plate prior to sectioning. (E) Sectioning of the FFPE block using the microtome. (F) Paraffin sections transferred in the RT water bath appear wrinkled (higher magnification, right). (G) Wrinkled sections placed in the 40°C water bath stretch propely before mounting. (H) SuperFrost™ Plus microscope slide showing two mounted FFPE organoid sections.

This section details 3 μm sections for histomorphological and immunohistochemical analyses. Serial sectioning allows comprehensive analysis across the top, middle, and bottom levels. Expect ∼20 sections from 10–15 d organoids; ∼30–40 from >20 d organoids. See Figure 6. Cool the FFPE block on a cooling plate at −15°C for 15min.

43. Set up the microtome (Figure 6A):
  a. Insert a new razor blade.
  b. Knife angle 8°.
  c. Align block holder.
  d. Trim mode 10μm.
  e. Place the waste tray.
44. Prepare two water baths: 20°C and 40°C (Figure 6B).
45. Insert the FFPE block into the holder. The organoid appears as a small white dot (Figure 6C).

***Note:*** Very small organoids (<400μm) may only become visible during trimming; if absent after several trims, the organoid may have been lost.

46. Trim 10μm until the organoid is visible on the cut surface (Figure 6E).
47. Return the block to the cooling plate for 10min (Figure 6D).

**CRITICAL:** Do not over-trim; once visible, proceed to fine sectioning. Switch to “Fine” mode and adjust the cutting thickness to 3μm.

48. Fine sectioning at 3μm.
49. Float sections in the 20°C water bath; mount on SuperFrost-Plus microscope slides (Figure 6F).
50. Transfer slides into a 40°C water bath; allow the section to flatten, remount (Figures 6G and 6H).
51. Dry slides at 40°C for at least 2h.
52. Store between 20°C–25°C.

### Deparaffinization

**Timing: 20 min**

53. Deparaffinize as follows:
  a. Immerse slides in xylene 2x 3min.
  b. Isopropanol 2x 3min.
  c. 96% isopropanol 1x 3min.
  d. 70% isopropanol 1x 3min.
  e. 50-% isopropanol 1x 3min.
  f. dH_2_O 2x 3min.

***Note:*** Handle xylene in a fume hood.

54. Keep slides in dH_2_O until further processing.

### Histomorphological characterization: H&E staining

**Timing: 20 min**

H&E provides a comprehensive morphological assessment. Hematoxylin stains nuclei deep blue-purple; eosin-phloxine counterstains cytoplasm and connective tissue pink-red (phloxine enhances cytoplasmic contrast).

**Figure 7:**
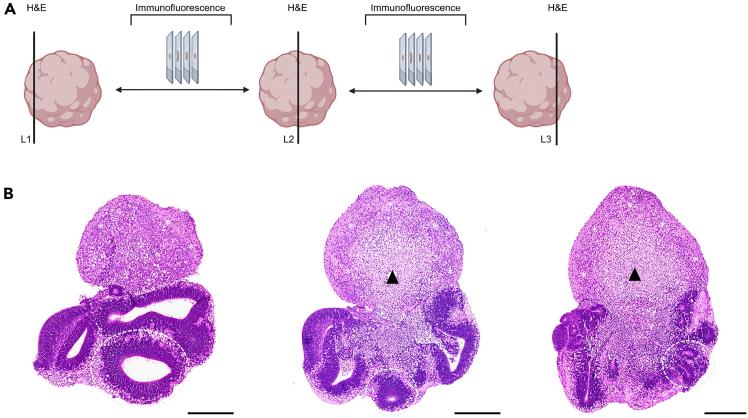
Serial sectioning approach for morphological evaluation of cerebral organoids (CO) (A) Schematic overview illustrating serial sectioning through the entire CO. Three representative levels—top (L1), middle (L2), and bottom (L3)—were selected at approx. 90 μm intervals for H&E staining to capture overall morphology. Intervening sections were reserved for immunofluorescence to enable detailed cell type-specific characterization and spatial analysis of differentiation. Created in BioRender. El-Debs, I. (2026) https://BioRender.com/g6d4380. (B) Representative H&E-stained sections of a 20-day-old CO at the three selected levels illustrating neuroepithelial rosettes (circles), cortical-like layer organization (stars), and overall viability including necrotic core formation (arrowheads) across the entire organoid. H&E images: 5×; Scale bars: 250μm.

**Figure 8:**
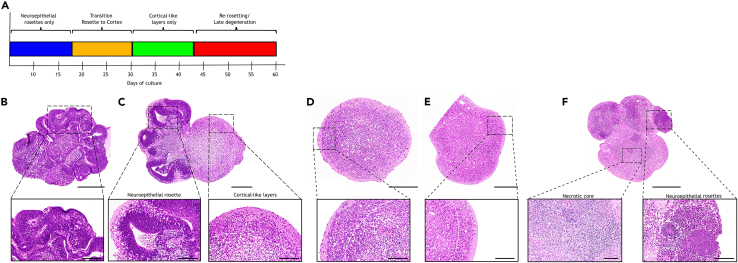
H&E staining of cerebral organoids (COs) at progressive maturation stages H&E overviews (5× and 30×); Scale bars: 250μm and 100μm. (A) Schematic illustration of CO maturation, highlighting the transition from neuroepithelial rosettes to cortical-like layers and eventual structural degeneration. (B) Day 10: immature CO with neuroepithelial buds containing numerous rosettes. (C) Day 20: immature CO with organized rosettes forming a ventricular zone (VZ, circle) together with cortical-like regions. (D) Day 30: rosettes have largely disappeared; cortical-like layers predominate. (E) Day 40: mature CO with well-defined stratified cortical-like architecture. (F) Day 50: CO showing rosettes (circles) and an enlarged necrotic core disrupting cortical organization.

**Figure 9:**
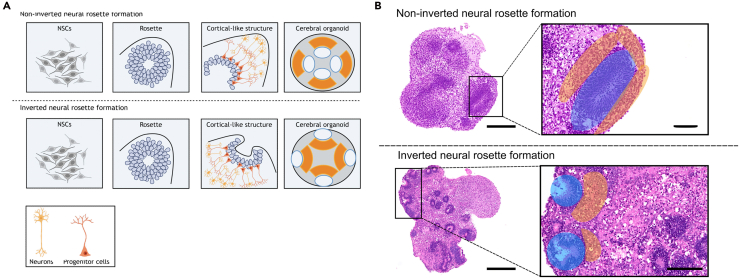
Schematic and histological representation of neuroepithelial rosette orientation in cerebral organoids (COs) (A) Schematic overview: Neural stem cells form neuroepithelial rosettes, which give rise to cortical-like layers. In non-inverted COs, differentiation occurs outward, with peripheral neuronal maturation and inward-facing rosettes. In inverted COs, cortical-like layers form inwardly, with peripheral rosettes. Hybrid COs exhibit both orientations, showing bidirectional differentiation. Created in BioRender. El-Debs, I. (2026) https://BioRender.com/vi1rrp3. (B) Representative H&E-stained sections of COs show non-inverted and inverted rosettes. Blue shading indicates rosette positions; orange shading highlights cortical-like layers. In non-inverted COs, rosettes face inward with peripheral cortical-like expansion. In inverted COs, rosettes are peripheral, with inward cortical-like formation. Partially inverted COs display mixed orientation features. H&E overviews (5× and 30×); Scale bars: 250μm and 100μm.

For structural analysis, three representative sections per organoid—top (L1), middle (L2), bottom (L3)—were used to capture morphology across the entire sample (schematic in Figure 7A). Morphological parameters included: neuroepithelial rosette number, size and orientation (non-inverted, partly inverted, inverted), presence of cortical-like layers, and viability (necrosis/structural disintegration). Representative examples are shown in Figures 7B, 8, and 9.

**CRITICAL:** Inadequate serial sectioning may miss key structures and misclassify differentiation. Analyze three levels (L1–L3); for smaller organoids (<400 μm), two levels are generally sufficient and preserve material for IF.

55. Incubate deparaffinized FFPE slides in Mayers‘ hematoxylin for 15 minutes.

***Note:*** Filter hematoxylin and eosin before use.

56. Rinse in cold tap water for 10 minutes until “blueing”.
57. Incubate in Eosin-Phloxine 3min.
58. Rinse with tap water for 3 minutes.
59. Dehydrate:
  a. 96% isopropanol 2min.
  b. 100% isopropanol 3x 2min.
  c. Xylene 3min.
60. Mount with Entellan and coverslip.

### Characterization of cellular composition and differentiation: Immunofluorescence staining

**Timing: 6 h to 2 days**

This section describes analysis of CO cellular architecture and differentiation using single- or double-color immunofluorescence with cell type-specific markers. IF complements H&E by resolving neural/glial subpopulations and their spatial arrangement within cortical-like layers. Key applications:

I) Viability (Caspase-3 for apoptosis, Ki-67 for proliferation);

II) Progenitor zones (SOX1, SOX2, PAX6, Nestin);

III) Intermediate progenitors (TBR2) and neuronal subtypes (TBR1, CTIP2, SATB2∗, NeuN);

IV) Neuronal networks (TUJ1);

V) Gliogenesis (GFAP, OLIG2);

VI) Cortical plate organization/polarity (Reelin).

∗SATB2 detection varies between organoids (see Figure S1).

**Figure 10:**
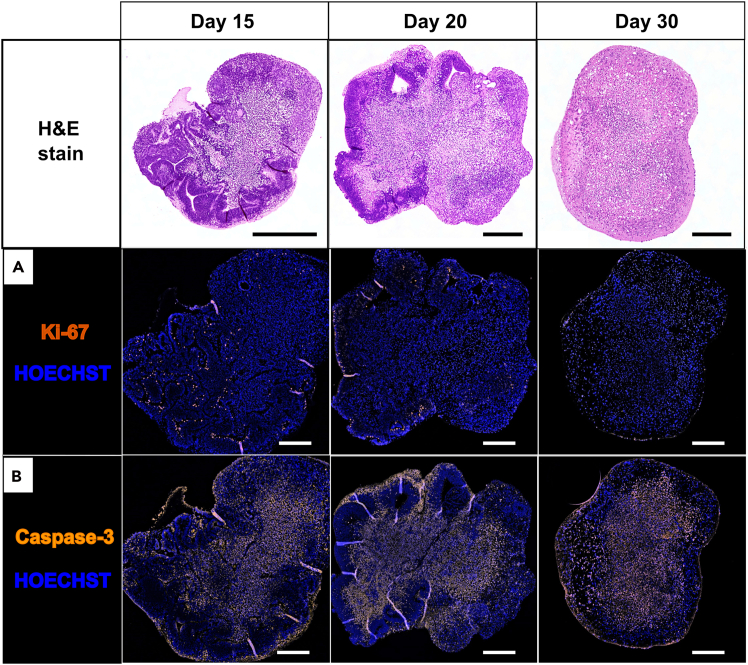
Immunofluorescence (IF) analysis of organoid viability at days 15, 20, and 30 Ki-67^+^ cells indicate proliferative activity mainly within neuroepithelial regions, while Caspase-3^+^ cells mark apoptotic activity predominantly in the organoid core. H&E overviews (5×) and IF overviews (10×). Scale bars: 250 μm.

**Figure 11:**
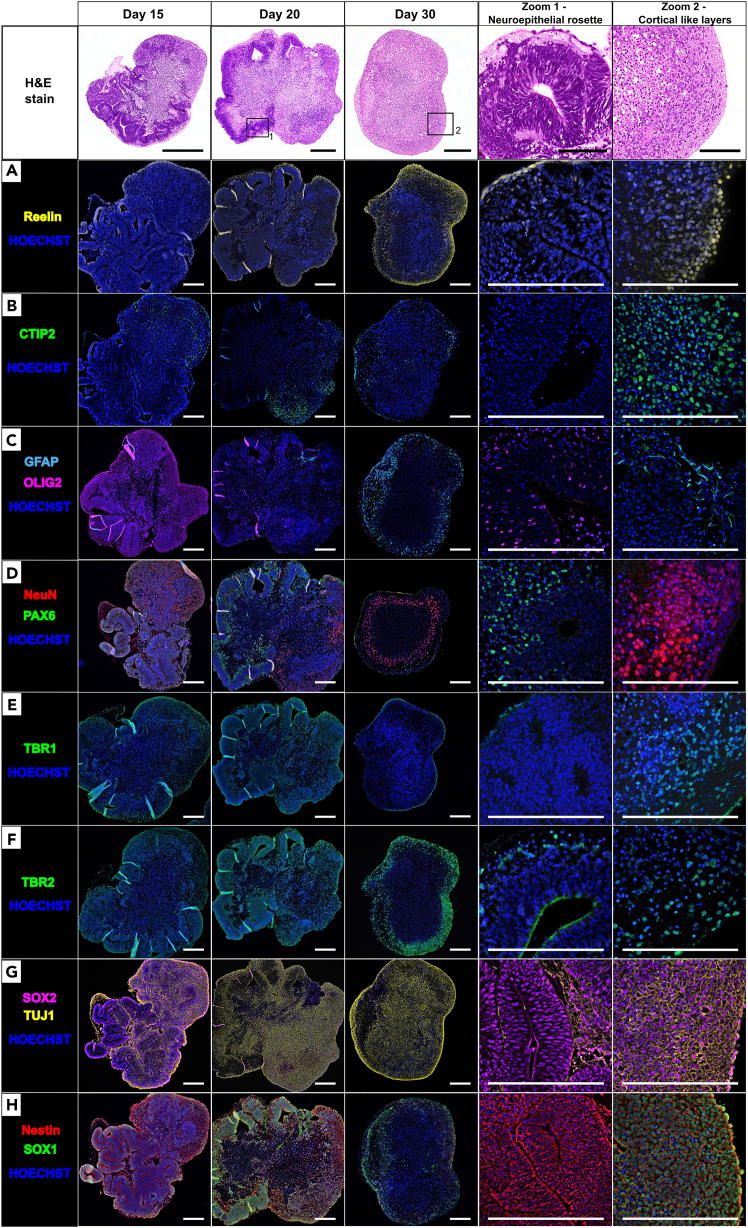
Immunofluorescence (IF) analysis of murine cerebral organoids (COs) at different maturation stages (days 15, 20, and 30) COs were maintained in cortical maturation medium (CMM) supplemented with B27. Representative IF images alongside H&E overviews (5×, 30×); IF (10×, 63×) show marker expression in neuroepithelial rosettes and cortical-like regions. Markers highlight: (A) cortical plate orientation (Reelin), (B) deep-layer neurons (CTIP2), (C) glial differentiation (OLIG2, GFAP), (D) progenitors and mature neurons (PAX6, NeuN), (E and F) early neurons and intermediate progenitors (TBR1, TBR2), and (G–H) progenitor/stem cell markers (SOX2, TUJ1, Nestin, SOX1). Together, these illustrate progressive neuronal and glial differentiation and cortical-like organization during maturation. Scale bars: 250 μm (for H&E zoom: 100μm).

Antibodies are listed in Table 1. Representative patterns across maturation are shown in Figures 10 and 11.

**Table 1:** Primary antibodies (target, localization, dilution, incubation)

Antibody	Target	Signal localization	Dilution	Incubation
BCL11b/CTIP2 (Invitrogen, # PA5-117429)	Neurons in layer V	Nucleus (+Cytosol)	1:1000	4°C; >12h
Cleaved caspase-3 (Cell Signaling Technology, # 9661)	Apoptotic cells	Cytosol (+Nucleus)	1:800	4°C; >12h
GFAP (ThermoFisher, # PA-110004)	Astrocytes	Cytosol	1:400	4°C; >12h
Ki-67 (Invitrogen, # MA5-14520)	Proliferative cells	Cytosol	1:100	4°C; >12h
NeuN (SYSY, #266004)	Postmitotic neurons	Nucleus	1:200	20°C–25°C; 1h or 4°C; >12h
Nestin (Invitrogen, # PA5-143578)	Intermediate filament in neural stem cells	Cytosol	1:1000	4°C; >12h
Olig2 (Abcam, # 109186)	Oligodendrocytes	Cytosol + Nucleus	1:50	4°C; >12h
PAX6 (Invitrogen, # 42-6600)	Radial glia cells	Nucleus	1:100	4°C; >12h
Reelin (Invitrogen, # PA5-78413)	Neurons at cortical plate + Cajal-Retzius cells	Cytosol + Nucleus	1:100	4°C; >12h
SATB2 (Invitrogen, # ZE4319632A)	Cortical neurons in layers II - IV	Nucleus (+Cytosol)	1:1000	4°C; >12h
SOX1 (Abcam, # 87775)	Neural stem cells	Nucleus	1:1000	4°C; >12h
SOX2 (Invitrogen, # 14-9811-82)	Multipotent neural stem cells and radial glia cells	Nucleus	1:100	4°C; >12h
TBR1 (Invitrogen, # YF3956298A)	(Corticothalamic-) Immature neurons	Nucleus	1:500	4°C; >12h
TBR2 (Invitrogen, # 720200)	Intermediate neuronal progenitor cells	Cytosol	1:500	4°C; >12h
TUJ1 (Invitrogen, # MA1-118)	Neuronal specific β-tubulin	Cytosol	1:200	4°C; >12h

#### Preparation and antigen retrieval

61. Deparaffinize as in step 53.
62. Antigen retrieval: immerse in citrate buffer, pH 6.0; heat in a steam/pressure(vapor) pot for 20min, cool between 20°C–25°C.

***Optional:*** Optimal conditions may vary by antibody.

63. Wash 2x in 1X PBS 2min.
64. Draw a hydrophobic barrier around sections (PAP/grease pen).

**CRITICAL:** Reapply barrier as needed to prevent drying/spillover.

***Note:*** Depending on the size of the sample area, the hydrophobic barrier can accommodate approximately 100–200 μL of antibody solution per slide.

#### Permeabilization and blocking

65. Add permeabilization buffer for 10min between 20°C–25°C.
66. Drain; block with 5% BSA in permeabilization buffer 1 h between 20°C–25°C.

#### Primary antibody incubation

67. Remove blocking buffer; add primary antibody, diluted in 1% BSA in 1x PBS (prepared from the 5% BSA/permeabilization buffer stock with PBS 1X).

**CRITICAL:** Use 1% BSA in PBS for both primary and secondary antibodies.

68. Incubate at least 12h at 4°C in a humidified chamber.

***Optional:*** See Table 1 for recommended times.

69. Next day, wash 2x in 1x PBS 2min each.
70. Permeabilization buffer 10min, drain.

#### Secondary antibody and nuclear staining

71. Add fluorophore-conjugated secondary antibody diluted in 1% BSA in 1x PBS; incubate 1 h at RT in the dark.
72. Wash 2x in 1 x PBS 2min each.
73. Nuclear stain with HOECHST.
  a. Dilute 2 μl of HOECHST in 40 ml 1x PBS (1:20.000).
  b. Incubate 5min in the dark.

***Note:*** DAPI is an alternative depending on instrument settings.

74. Wash 2x in 1x PBS 1X, 2min each.
75. Incubate with Washing buffer 2 10min in the dark.
76. Briefly rinse with dH_2_O.
77. Mount with Shandon™ Immo-Mount (avoid air bubbles).

***Note:*** Medium polymerizes within ∼20 min; no additional sealing of coverslip required.

## Expected outcomes

This protocol provides a robust and time-efficient method for generating mature murine cerebral organoids (COs) from embryonic stem cells (E14.5). Once EBs have formed, COs can be matured for up to 40–60 days. CMM + B27/SM1 supports long-term viability, neurogenesis, and progressive differentiation beginning around day 20. Even small organoids (<400 μm) can be fixed in 4% paraformaldehyde or 10% buffered formalin and processed into serial 3 μm thin sections. H&E plus IF enables comprehensive analysis of organoid cytoarchitecture, cellular identity, and maturation state.

### Early (days 10–20)

Days 10–15, nearly all organoids contain neuroepithelial rosettes (87%–78%) composed of SOX1^+^, SOX2^+^, PAX6^+^ progenitors and Nestin^+^ precursors, representing ventricular zone (VZ)-like structures. Rosette inversion is observed in ∼40% of organoids. Differentiation is minimal, with organoids dominated by progenitor-rich zones. By day 20, rosettes decline (52%), and 32% of organoids already display cortical-like layers. TUJ1^+^/TBR1^+^ neurons emerge adjacent to rosettes, and TBR2^+^ intermediate progenitors populate a developing subventricular zone (SVZ). Necrosis increases (84%), but organoids remain viable.

### Transition to maturation (days 25–35)

Days 25–30: rosettes further diminish (22% at day 25; 6% at day 30), while cortical-like layers predominate (78%–88%). By day 30, CTIP2^+^ and TBR1^+^ neurons populate deeper cortical-like layers, NeuN^+^ mature neurons migrate into upper layers, and Reelin^+^ cells mark laminar organization. Necrotic cores are present in all organoids; some organoids degenerate completely (∼12%). By day 35, rosettes are absent, leaving exclusively differentiated cortical-like layers (100%). SATB2^+^ upper-layer neurons appear variably from day 30 (Figure S1). GFAP^+^ astrocytes emerge, indicating gliogenesis.

### Late (days 40–60)

Day 40: organoids reach diameters >2 mm and display cortical-like layered organization, although all exhibit necrotic cores. Rosette-like structures are observed sporadically between days 45–55 (20%–50%), reflecting heterogeneity in maturation dynamics and potentially representing either persistent or newly emerging progenitor-associated architectures. OLIG2^+^ precursor cells are detectable at earlier developmental stages (e.g., day 15) but gradually decline by day 30 as the organoids mature. This reduction in OLIG2 expression is consistent with the expected downregulation of this early progenitor-associated marker as cells differentiate into more mature phenotypes. In contrast, astrocytic populations continue to increase over time. By days 55–60, > 50% are non-viable despite ongoing differentiation processes.

Overall, this culture system recapitulates key aspects of early murine corticogenesis in vitro, including progenitor zone organization, neuronal subtype specification (deep/upper layers), gliogenesis, and the transition from rosette-rich states toward stratified cortical-like architecture.

## Limitations

COs inherently display biological and morphological heterogeneity in size, shape, and rosette organization. Notably, two rosette polarities— “apical-in” and “apical-out”—were originally described and quantified in 3D ESC cultures; and extracellular matrix composition (e.g., laminin) has been shown to influence rosette orientation and polarity consistency.⁠^5^ Our observations of inward-, outward-, and partly inverted rosette arrangements (often coexisting with cortical-like layers at early–mid stages) are consistent with these prior findings and likely reflect ECM exposure and local mechanical cues during culture. Small multilayered rosettes may be present beyond ∼40 days, consistent with heterogeneous maturation trajectories. Because organoids were analyzed only at fixed timepoints rather than followed longitudinally, it remains unclear whether these late rosettes reflect de novo formation or the persistence of earlier structures. The occurrence may also indicate the presence of residual stem or progenitor cell reservoirs capable of generating neuroepithelial rosettes in a subset of mature organoids.

Expression of late markers such as SATB2 also varies between organoids (see Figure S1), underscoring intrinsic variability. While such diversity reflects the self-organizing nature of organoids and provides insights into alternative developmental pathways, it may limit direct comparability across samples.

A central limitation of prolonged culture is the necrotic core due to diffusion constraints and lack of vascularization. As organoids expand beyond 2 mm in diameter (typically after 40–50 days), central necrosis increases despite trophic supplementation and orbital agitation. We therefore recommend limiting cultivation to 40 days to ensure reliable histological and molecular characterization before necrosis becomes predominant. Although prolonged culture up to 60 days is technically feasible, it increases heterogeneity, reduces viability, and limits suitability for standardized downstream applications. Minor variation in organoid numbers per time point reflects differences in survival and occasional sample loss during tissue processing. As part of method optimization, we also evaluated alternative approaches commonly applied in human dorsal cerebral organoid protocols, including several days of dual SMAD inhibition and omission of Matrigel. Neither modification improved reproducibility, maturation, or overall organoid quality relative to the conditions outlined here.

Regarding tissue processing, the FFPE workflow combined with microtome serial sectioning offers robust long-term storage stability and produces thin, uniform suitable for high-resolution morphological assessment. FFPE blocks are also relatively cost-efficient to archive. However, the workflow is more time-consuming due to fixation, dehydration, embedding and several sectioning steps. In contrast, cryopreservation of organoids in OCT/sucrose followed by cryo-sectioning is generally faster, primarily because thicker sections (approximately 30 μm) shorten processing time. These thicker sections, however, reduce morphological overview and structural resolution. Cryo-based workflows additionally require dedicated low-temperature storage, and long-term stability of cryopreserved samples is generally lower to that of paraffin-embedded material.

Finally, while IF confirms the presence of progenitors, immature/mature neurons, astrocytes, and oligodendrocytes, microglia are absent under standard conditions. Future refinements such as microglial co-culture or vascularization strategies may enhance cellular complexity and increase the translational relevance of the model.

## Troubleshooting

### Problem 1

Insufficient EB formation or multiple EBs in a well (see step 9).

### Potential solution

EB formation usually initiates within 12 h. Absence at 24 h suggests poor health or over-confluency of ESCs. Verify ESC morphology by brightfield microscopy. Multiple EBs per well do not necessarily impair organoid formation, but smaller aggregates may disintegrate during transfer (Figure 2C). Reduce initial seeding density to favor single EB aggregation.

### Problem 2

Premature Matrigel polymerization; insufficient EB coating (see step 29).

### Potential solution

Keep Matrigel on ice and pre-cool tips. Insert the tip deeply (slightly bend) before dispensing to ensure complete coverage. Incomplete coating disrupts neuroepithelium and growth (Figures 3 and 4).

### Problem 3

Uneven organoid growth; some fail to enlarge (see step 35).

### Potential solution

Start with similar-sized organoids. Avoid >12 organoids/dish; transfer >1 mm organoids to fresh dishes or individual wells. Use fresh medium. Lack of growth over time likely indicates non-viability—exclude such organoids.

### Problem 4

Heterogeneous morphology and development (see step 38).

### Potential solution

Standardize ESC passage (e.g., 10–20), ensure accurate seeding density, pre-test/choose consistent Matrigel lots, and maintain uniform pipetting technique.

Other approaches intentionally avoid Matrigel embedding, thereby reducing variability associated with extracellular matrix composition and handling. Guided differentiation strategies using defined patterning factors, for example, BMP7 based protocols generating thalamic organoids, have been shown to produce highly uniform neural spheroids with consistent regional identities.⁠^6^ Similarly, oligodendrocyte-enriched brain organoid protocols demonstrated that Matrigel-free, suspension-based cultivation can yield organoids with improved structural reproducibility and reduced batch-to-batch variability while still supporting complex glial and neuronal maturation.⁠^7^ These guided, matrix-free approaches represent valuable alternatives for researchers prioritizing homogeneity and standardized lineage specification.

### Problem 5

Loss of neuroepithelial rosettes after day 30.

### Potential solution

Expected developmental transition toward cortical-like layers. If rosette analysis is required, fix ≤day 25.

### Problem 6

Organoids are not visible during embedding or in the FFPE block.

### Potential solution

Eosin-phloxine can fade during dehydration. Use 1.5 ml tubes to aid sedimentation; leave a small residual volume during exchanges to avoid aspiration. Process multiple organoids on a dark background to enhance visibility and reduce loss (Figure 5B). Very small organoids may become visible only during trimming; proceed carefully, then section at 3 μm.

### Problem 7

Difficulty identifying small organoids during embedding (see step 40).

### Potential solution

Process multiple small organoids on a dark background. Clearly label tubes, and minimize residual liquid during solution changes to prevent accidental loss. Pooling small organoids for embedding can also reduce material loss.

### Problem 8

Tissue sections tear/collapse during microtome sectioning (see step 46).

### Potential solution

If the region of interest is intact, the section remains usable. Otherwise, re-section. Reduce cutting speed, ensure the block is well chilled, use a sharp blade, and confirm proper trimming/orientation.

## Resource availability

### Lead contact

Further information and requests for resources and reagents should be directed to the lead contact, Dr. Julia Sehl-Ewert (julia.sehl-ewert@fli.de).

### Technical contact

Technical questions on executing this protocol should be directed to and will be answered by the technical contact, Issam El-Debs (issam.el-debs@fli.de).

### Materials availability

This study did not generate new, unique reagents.

### Data and code availability

This study did not generate any datasets or codes.

## Acknowledgments

We would like to thank Silvia Schuparis and Robin Brandt for their continuous assistance and support during sample processing. Furthermore, we sincerely thank Angele Breithaupt, Lukas Mathias Michaely, Tobias Britzke, and Viktoria Korff for their valuable contributions and scientific advice throughout the course of this work. The project was funded by the DURABLE-Research Network against Epidemics (grant no. 101102733). The graphical abstract and schematic illustrations were created using BioRender.com.

## Author contributions

I.E.-D. designed, implemented, and wrote the protocol. T.C.M., M.R.K., and J.O.M. participated in method development and revised the manuscript. J.S.-E. conceived, designed, and supervised the study and revised the manuscript.

## Declaration of interests

The authors declare no competing interests.
