# Supplementary material for: Protocol for the establishment and morphological characterization of long-term cultivated murine cerebral organoids
Source: STAR Protoc. 2026 Jan 9;7(1):104324. doi: 10.1016/j.xpro.2025.104324 (PMC12819030; doi:10.1016/j.xpro.2025.104324)
Supplement: Document S1. Figure S1 [file mmc1.pdf]

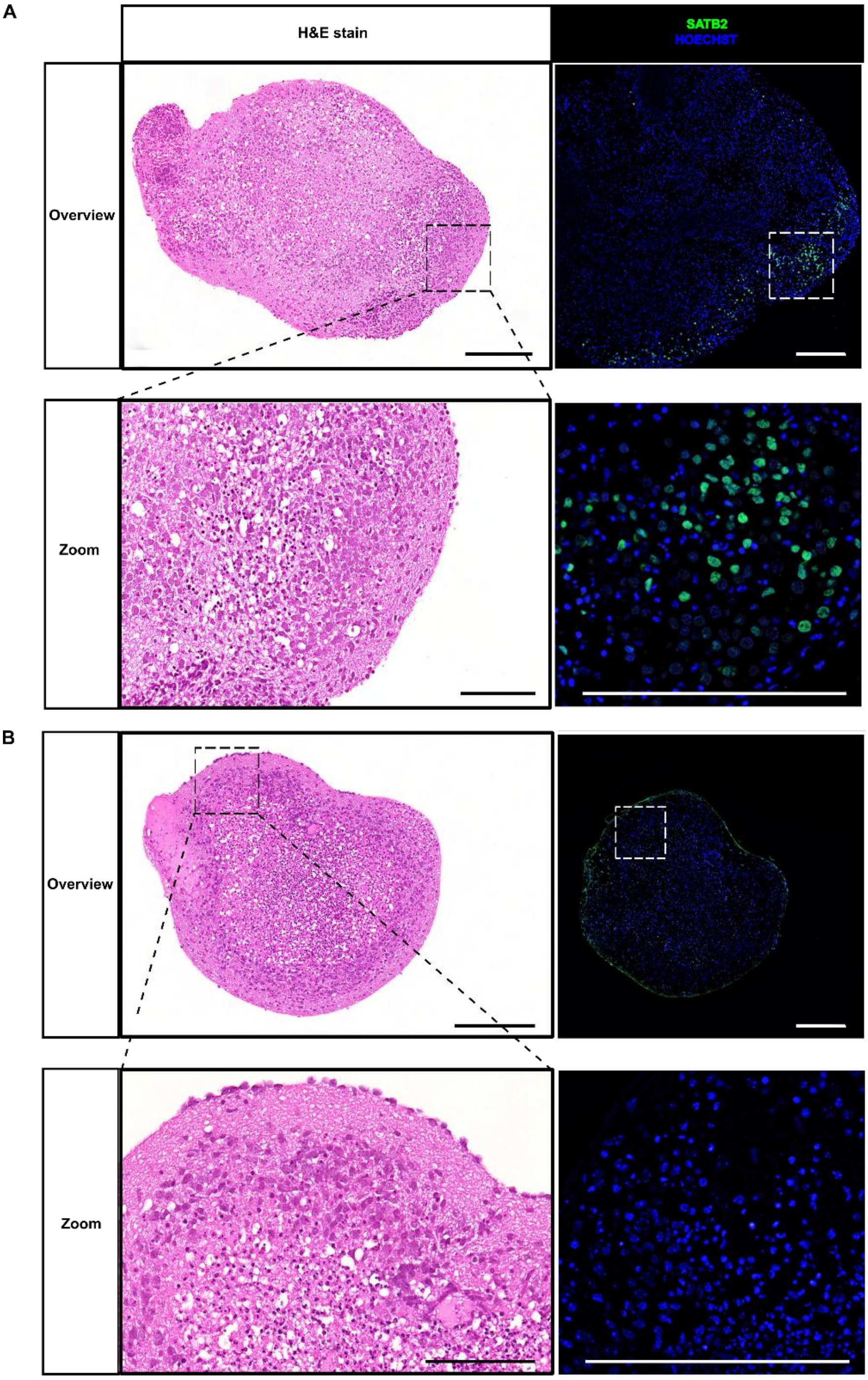

Fig S1. Immunofluorescence (IF) analysis of variable SATB2⁺ expression of upper-layer cortical neurons in mature 30 days old cerebral organoids (COs), related to step 67. (A) Dense $SATB2^{+}$ expression within cortical-like layers; (B) Absence of $SATB2^{+}$ expression in cortical neurons. H&E overviews (5×, 30×) and IF overviews/zooms (10×, 63×). Scale bars: 250µm and 100µm.
